# A Two-Year Randomized Trial of Interventions to Decrease Stress Hormone Vasopressin Production in Patients with Meniere’s Disease—A Pilot Study

**DOI:** 10.1371/journal.pone.0158309

**Published:** 2016-06-30

**Authors:** Tadashi Kitahara, Hidehiko Okamoto, Munehisa Fukushima, Masaharu Sakagami, Taeko Ito, Akinori Yamashita, Ichiro Ota, Toshiaki Yamanaka

**Affiliations:** 1 Department of Otolaryngology, Nara Medical University, Kashihara-city, Nara, Japan; 2 Department of Otolaryngology, Osaka Rosai Hospital, Sakai-city, Osaka, Japan; 3 Department of Physiology, Okazaki Research Institute, Okazaki-city, Aichi, Japan; Ohio State University, UNITED STATES

## Abstract

Meniere's disease, a common inner ear condition, has an incidence of 15–50 per 100,000. Because mental/physical stress and subsequent increase in the stress hormone vasopressin supposedly trigger Meniere's disease, we set a pilot study to seek new therapeutic interventions, namely management of vasopressin secretion, to treat this disease. We enrolled 297 definite Meniere's patients from 2010 to 2012 in a randomized-controlled and open-label trial, assigning Group-I (control) traditional oral medication, Group-II abundant water intake, Group-III tympanic ventilation tubes and Group-IV sleeping in darkness. Two hundred sixty-three patients completed the planned 2-year-follow-up, which included assessment of vertigo, hearing, plasma vasopressin concentrations and changes in stress/psychological factors. At 2 years, vertigo was completely controlled in 54.3% of patients in Group-I, 81.4% in Group-II, 84.1% in Group-III, and 80.0% in Group-IV (statistically I < II = III = IV). Hearing was improved in 7.1% of patients in Group-I, 35.7% in Group-II, 34.9% in Group-III, and 31.7% in Group-IV (statistically I < II = III = IV). Plasma vasopressin concentrations decreased more in Groups-II, -III, and -IV than in Groups-I (statistically I < II = III = IV), although patients’ stress/psychological factors had not changed. Physicians have focused on stress management for Meniere’s disease. However, avoidance of stress is unrealistic for patients who live in demanding social environments. Our findings in this pilot study suggest that interventions to decrease vasopressin secretion by abundant water intake, tympanic ventilation tubes and sleeping in darkness is feasible in treating Meniere’s disease, even though these therapies did not alter reported mental/physical stress levels.

***Trial Registration*:** ClinicalTrials.gov NCT01099046

## Introduction

Meniere's disease, characterized by recurrent vertigo, fluctuating hearing loss and persistent tinnitus, is a common disease with an incidence of 15–50 per 100,000 [1]. It has been reported that Meniere's disease is usually triggered by various kinds of stimuli, i.e. genetic, infectious, vascular, dietary, allergic, autonomic, endocrine, autoimmune, or other insults to the inner ear, associated with a small misplaced malfunctioning endolymphatic sac [2]. In spite of these undetermined insults, so many ENT doctors have had an impression of strong relationships between stress and Meniere's disease. This disease is supposedly triggered by mental and/or physical stress caused by participating in stressful daily activities and by interactions within stressful social environments. Several studies have reported psychological and cognitive therapies for Meniere’s disease [3]. Many physicians advise their patients to take adequate time out for stress management. However, it is difficult, indeed often unrealistic, for most of Meniere’s patients to undertake long-term psychological treatment and/or change their work situation to reduce stress in their daily life.

Temporal bone studies in 1938 were the first to show the otopathology in Meniere’s disease is inner ear hydrops [4,5]. Since then, others have reported that a disorder in water metabolism-related molecules causes this condition [6,7]. Our recent studies showed that plasma concentrations of the stress hormone vasopressin (pAVP), its receptor V2 (V2R) and V2R-linked water channel aquaporin-2 (AQP2) in the endolymphatic sac are significantly higher in Meniere's patients than in those without this disease [8,9]. Increases in pAVP caused by stress and subsequent activation of the V2R-AQP2 intracellular signaling cascade and endosomal trapping of AQP2 in the endolymphatic sac might underlie the pathogenesis of the inner ear hydrops that results in the intermittent vertigo, hearing loss and tinnitus characteristic of Meniere's disease [9].

In light of the above, the present pilot study provides a good start of scientific basis for proposing the new therapeutic strategy of management of vasopressin secretion for stress-related inner ear hydrops. This form of management is realistic and appropriate for patients living in present-day unstable and demanding social environments. In the present study, we proposed three kinds of interventions, i.e. abundant water intake, tympanic ventilation tubing and sleep in darkness, to possibly decrease stress hormone vasopressin production in patients with Meniere’s disease, and would like to discuss each feasibility and mechanism.

## Materials and Methods

This clinical study was registered with ClinicalTrials.gov (identification number: NCT01099046). The use of all the patients’ data in the present study was approved not only by the patients as written consents but by the Ethics Committee of Osaka Rosai Hospital (identification number: 2263).

### Patients

**Fig 1:**
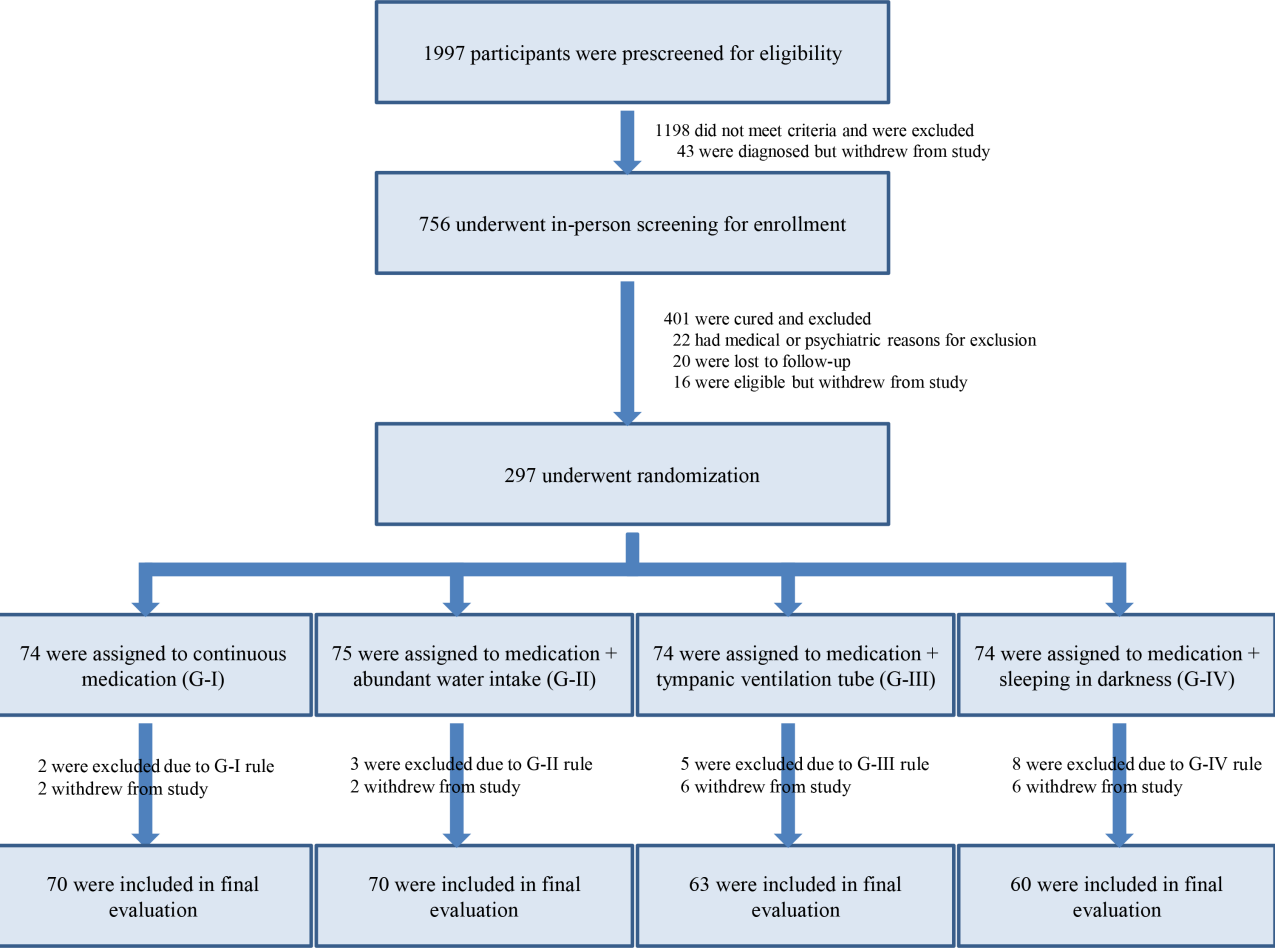
Flow chart of the present randomized controlled study.

The flow chart in Fig 1 depicts the screening, randomization and assessments of study participants performed in the present study.

Because of lack of the similar exploratory studies for treatments of Meniere’s disease, we did not do the power analysis for the appropriate sample size in this study. Therefore, we determined the study period enough to collect patients with vertigo in this pilot study. Between January, 2010 and December, 2012, 1997 patients with vertigo aged at least 20 years were prescreened for eligibility in the vertigo and dizziness department of Osaka Rosai Hospital to determine whether they had Meniere's disease according to the 1995 American Academy of Otolaryngology–Head and Neck Surgery (AAO-HNS) criteria [10]. Briefly, these criteria are as follows: (i) Repeated attacks of spontaneous vertigo lasting ≥ 20 minutes, if observed, accompanied by mixed type nystagmus. (ii) Fluctuating cochlear symptoms; a couple of hearing tests usually reveal marked fluctuation of the threshold in the low and middle tone range. A glycerol test or electrocochleogram is performed to detect endolymphatic hydrops, if necessary [11]. (iii) Exclusion of other causes. To rule out other disorders, thorough history-taking, neurological, neurotological and MRI examinations are performed, if necessary.

Definite Meniere’s disease was diagnosed in 756 patients who were then further screened for enrollment into the present study. Eligible patients were those in whom 3–6 months of basically fixed forms of medical treatment had produced insufficient benefit (i.e. recurrent vertigo attacks and/or no improvement in sensorineural hearing loss) and for whom surgical treatment would otherwise be considered. Thus, according to the treatment algorithm for Meniere’s disease described by Sajjadi and Paparella in a Lancet Seminar [12], interventions to decrease stress hormone vasopressin secretion were situated between medical and surgical treatments. Medical treatments were fixed basically including diuretics, betahistine, diphenidol, dimenhydrinate, and diazepam, all considered effective for persistent symptoms of Meniere’s disease [13].

### Randomization and Treatments

This clinical study is a randomized-controlled and open-label trial with four arms including a control group. Before randomization and assignment, electrocardiography, laboratory tests, oto-microscopy and questionnaires were used to identify and exclude patients with cardiac disease, renal dysfunction, middle ear problems and nyctophobia who cannot sleep in darkness. The remaining 297 definite Meniere's patients were randomly assigned through computer-generated block randomization organized by the clinical study section at Osaka Rosai Hospital to one of four treatment groups. Group-I (G-I, 74 patients, controls) continued to receive traditional oral medication as described above [13]. Group-II (G-II, 75 patients) received both medication and abundant water intake: 35 mL/kg/day as specified by a previous study [14] with a self-check diary of water volume/day. Group-III (G-III, 74 patients) received medication and had ventilation tubes inserted through an incision in their tympanic membranes under local anesthesia to relieve inner ear hydrops [15]. Group-IV (G-IV, 74 patients) received both medication and advice to sleep in darkness to maintain the hormonal circadian rhythm, defined as lying in bed in an unlit room less than 0.1 lux for 6–7 hours per night [16] with a self-check diary using an illuminometer (Panasonic Corp., Osaka, Japan). According to the questionnaires at randomization and assignment, there were no significant differences in the ratios of the number of cases with abundant water intake of more than 35 mL/kg/day habitually as treatments in G-II (n = 6 in G-I; n = 6 in G-II; n = 6 in G-III; n = 7 in G-IV) or those with sleep enough in darkness less than 0.1 lux habitually as treatments in G-IV (n = 7 in G-I; n = 7 in G-II; n = 6 in G-III; n = 7 in G-IV) among the four groups.

All patients were followed up for vertigo and hearing at least once a month for at least 24 months, until December, 2013. At their monthly check-ups, patients were excluded if, according to a self-declaration visual analogue scale, they had been < 75% compliant with the rules for their group: medication in G-I-IV; abundant water intake in G-II; sleep enough in darkness in G-IV. G-III patients were excluded if their ventilation tubes had been removed. Each patient was checked up only with each group’s rule. By the final evaluation 24 months after start of treatment, 70 cases remained in G-I, 70 in G-II, 63 in G-III, and 60 in G-IV (i.e. 4 in G-I, 5 in G-II, 11 in G-III and 14 in G-IV were excluded).

Throughput the study, intravenously administered anti-nausea (n = 9 in G-I; n = 9 in G-II; n = 8 in G-III; n = 8 in G-IV) and sedative drugs (n = 9 in G-I; n = 8 in G-II; n = 8 in G-III; n = 8 in G-IV) for vertigo attacks and oral corticosteroids (n = 7 in G-I; n = 7 in G-II; n = 6 in G-III; n = 7 in G-IV) for acute sensorineural hearing loss were added as required in all groups.

### Vestibular and Auditory Assessments

Vertigo lasting more than 20 minutes was regarded as a Meniere’s vertigo attack as specified by the 1995 AAO-HNS criteria [10]. Pre-admission frequency of vertigo was defined as the number of such attacks during the 6 months before admission to this study and post-treatment frequency of vertigo as the number of attacks during the 6 months between 18–24 months after admission. “Complete” control of vertigo (no vertigo) was defined as no vertigo attacks during the latter period. A ratio of pre-/post-treatment values of ≤ 0.8 was regarded as “better”, of ≥ 1.2 as “worse” and the remainder as “no change”.

Hearing was measured by pure tone audiometry and evaluated based on a 4-tone average calculated thus: (a+b+c+d)/4 (a, b, c and d being hearing at 0.5 kHz, 1 kHz, 2 kHz and 4 kHz, respectively) according to the modified 1995 AAO-HNS criteria. The worst hearing level during the 6 months before admission to this study was considered the pretreatment hearing level and the worst hearing level between 18–24 months the post-treatment hearing level. More than 10 dB differences in hearing levels before and after treatment were regarded as “better”, less than −10 dB differences as “worse” and the remainder as “no change”.

### Laboratory Tests and Questionnaires

Blood samples were collected between 8.00am-10.00am during remission of vertigo to minimize effects of circadian variation. Blood for pAVP assay was transferred into an ethylene-diamine-tetraacetic acid tube, centrifuged at 4°C and the separated plasma stored at −80°C. pAVP [17] and serum cortisol [18] were analyzed by radioimmunoassay.

Stress and psychological factors were evaluated by laboratory tests and questionnaires at the time of admission and at 24 months in all groups. Laboratory exams included stress-related molecules, plasma vasopressin (pg/mL) and serum cortisol (microg/mL). Questionnaires included the Self-rating Depression Scale (SDS) [19] and Stress Response Scale-18 (SRS-18) [20].

Patients with SDS scores >40 (possible range 20–80) were classified as having depression. The SDS consists of 10 positively and 10 negatively worded items that enquire about symptoms of depression. These scores were used to define categories of depression: not having significant depression (≤40 points); having significant depression (≥41 points). The SDS has been translated into Japanese and the validity of the Japanese version confirmed [19].

In this study, patients with SRS-18 scores >20 (possible range 0–54) were classified as having high stress. The SRS-18 consists of 18 items that enquire about stressful feelings due to stressful lifestyle. These scores were used to define categories of stressful feelings: not having significant stress (0–20 points); having significant stress (≥21 points). The SRS-18 has been published in Japanese and embodies a new concept of psychological measurement [20].

### Statistical Analysis

**Fig 2:**
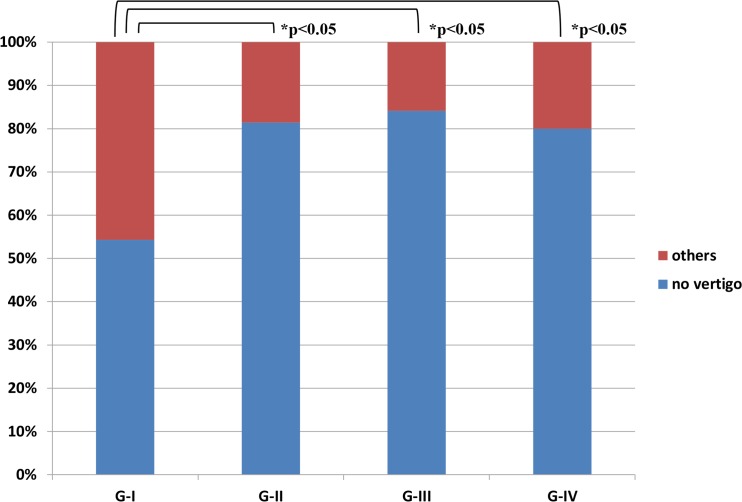
Vertigo attacks in patients with definite Meniere's disease 18–24 months into study. Ratios of the number of cases with “no vertigo” and “others”18–24 months after treatment are shown in each group. “No vertigo” means an absence of vertigo attacks from 18–24 months; “others” means better, worse and no change (as defined in Patients and Methods) during the same period. *: statistically significant. Percentages mean ratios of the number of these patients.

**Fig 3:**
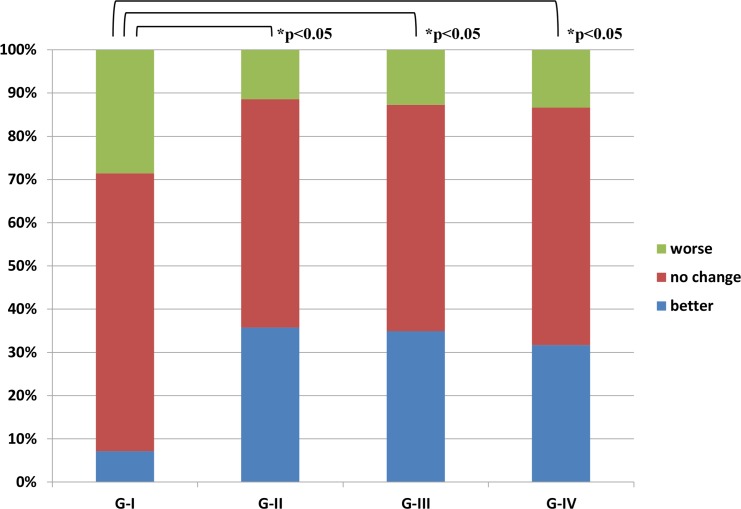
Two-year follow-up hearing in patients with definite Meniere's disease. Ratios of the number of cases with “better hearing”, “no change of hearing” and “worse hearing”18–24 months after treatment are shown in each group. “Better”, ≥10 dB difference between pre- and post-treatment hearing levels; “worse”, ≤−10 dB difference; “no change”, other. *: statistically significant. Percentages mean ratios of the number of these patients.

**Fig 4:**
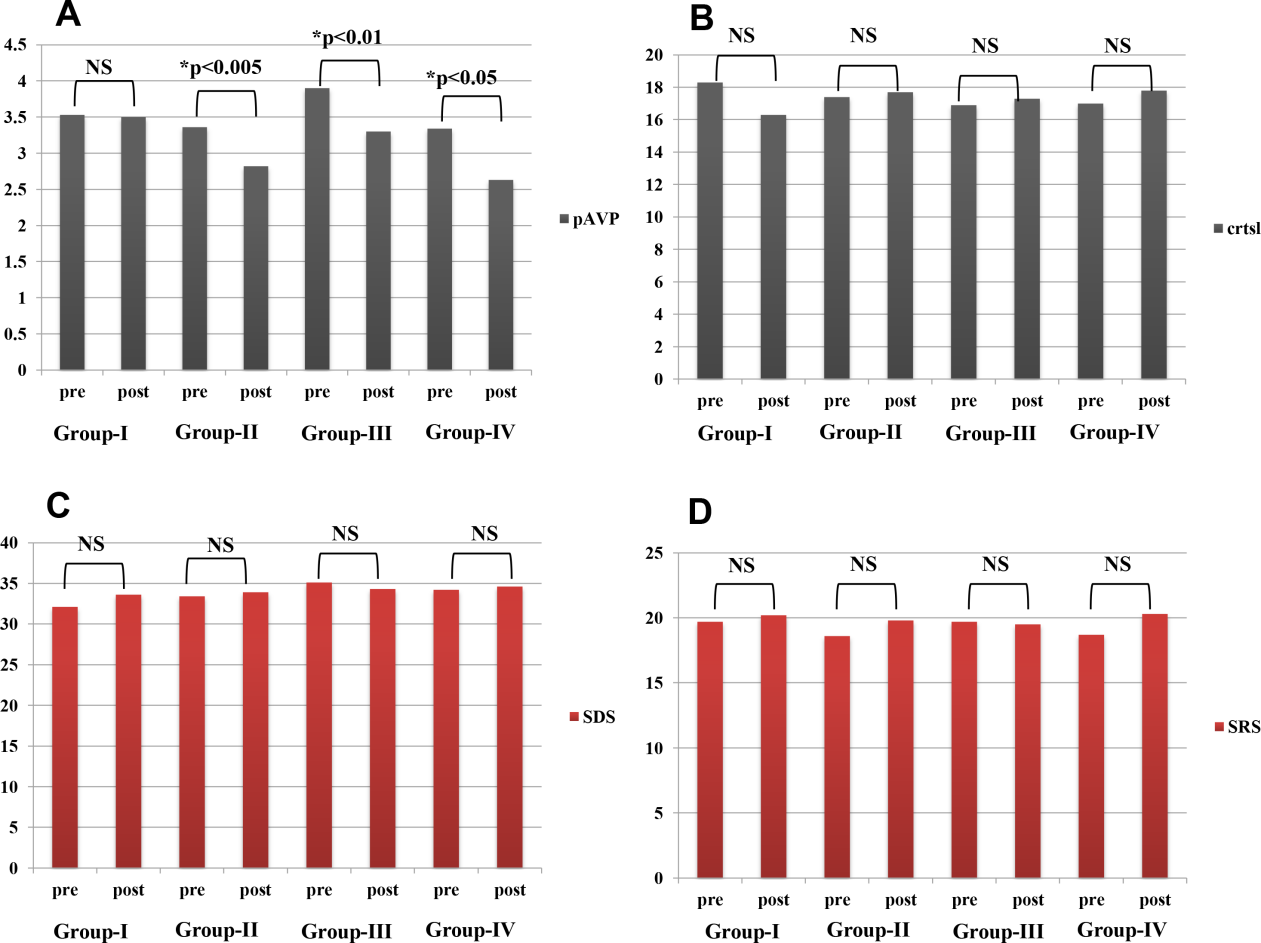
Pre-and post-treatment stress and psychological assessment in patients with definite Meniere's disease. In all four panels, averaged levels or points were compared between pre- and post-treatments in each group. Panel A: In comparison with G-I, plasma vasopressin concentrations (pAVP: pg/mL) were significantly reduced in G-II, G-III and G-IV. Panel B: Serum cortisol concentrations (crtsl: μg/mL) did not change. Panel C: Self-rating depression scale scores (SDS: points) did not change. Panel D: Stress response scale scores (SRS-18: points) did not change. *: statistically significant; NS: no statistical significance.

All treatment results were expressed as ratios of the number of cases and assessed statistically by SPSS version 16.0 (Chicago, IL). For post-treatment results, the χ^2^ test for vertigo in Fig 2 and Mann-Whitney U-test for hearing in Fig 3 were used to compare various pairs of the four groups, G-I, G-II, G-III, and G-IV. Repeated measures ANOVA were used to examine the statistical significance of changes in laboratory data in Fig 4A and 4B and questionnaire points in Fig 4C and 4D after each treatment. All reported p-values were two-sided and those under 0.05 were considered significant.

We did not do the power analysis for the appropriate sample size in the present pilot study as mentioned above. Therefore, we interpreted the present pilot data as feasibility/direction rather than efficacy/effectiveness.

## Results

Relevant data of patients with definite Meniere’s disease are shown in Table 1. There were no significant differences in these variables among the four groups studied.

**Table 1:** Materials consist of patients with definite Meniere's disease in Groups I, II, III and IV. Sex (male/female); Age, age (yr) at start of treatment; Lat, laterality (left/right); Duration, duration of disease (months) before start of treatment; pAVP, plasma vasopressin concentration (pg/mL) at start of treatment; crtsl, serum cortisol concentrations (μg/mL) at start of treatment; SDS, self-rating depression scale score at start of treatment; SRS-18, stress response scale score at start of treatment; Vertigo, average number of definitive spells per month during the 6 months before start of treatment; Hearing: worst average of hearing level during the 6 months before start of treatment. There were no significant differences between the four groups in these variables (n.s.).

characteristic	medication only	medication+water	medication+tube	medication+sleep	statistics
	n = 70	n = 70	n = 63	n = 60	
Sex (m/f)	25/45	28/42	22/41	22/38	n.s.
Age (yr)	49.7+16.5	46.7+13.7	51.7+14.7	49.7+11.9	n.s.
Lat (l/r)	39/31	36/34	32/31	32/28	n.s.
Duration (mo)	30.3+22.7	32.1+22.2	37.2+20.9	33.8+23.2	n.s.
pAVP (pg/ml)	3.5+3.2	3.4+2.7	3.9+2.7	3.3+3.1	n.s.
crtsl (microg/ml)	18.3+7.5	17.4+7.5	16.9+7.2	17+7.2	n.s.
SDS (point)	32.1+9.1	33.4+9.4	35.1+9.6	34.2+9.8	n.s.
SRS-18 (point)	19.7+6.3	18.6+6.3	19.7+6.4	19.2+6.3	n.s.
Vertigo (/mo)	1.6+1	1.7+1	1.8+1.1	1.7+1.1	n.s.
Hearing (dB)	47.3+13.4	48.7+14.9	49.5+13.2	49.1+15.1	n.s.

Vertigo control and hearing improvement according to the criteria of the 1995 AAO-HNS [10] during the 2 years of follow-up are shown in Fig 2 and Fig 3. As shown in Fig 2, 54.3%, 81.4%, 84.1% and 80.0% of G-I, G-II, G-III and G-IV patients, respectively, had no vertigo attacks during the 18–24 month period. These results were statistically significant as follows: G-I < G-II = G-III = G-IV (χ^2^ test: p<0.05 between G-I and the others). As shown in Fig 3, in the G-I group hearing had improved (i.e. better by ≥10 dB) in 7.1% of patients and worsened (i.e. worse by ≤−10 dB) in 28.6% by the end of the 2-years of follow-up. During the same period, hearing in G-II improved in 35.7% and worsened in 11.4%, in G-III improved in 34.9% and worsened in 12.7%, and in G-IV improved in 31.7% and worsened in 13.3%. These results are statistically significant as follows: G-I < G-II = G-III = G-IV (Mann-Whitney U-test: p<0.05 between G-I and the others).

Regarding stress and psychological factors, in comparison with the G-I group (3.53±3.18→3.50±3.03, p = 0.887), pAVP concentrations (pg/mL) were significantly reduced after each treatment in G-II (3.36±2.71→2.82±2.81, p = 0.003), G-III (3.90±2.72→3.30±2.26, p = 0.008), and G-IV (3.34±3.12→2.63±1.54, p = 0.026) (Fig 4A). However, plasma cortisol concentrations (μg/mL) did not change in any group (G-I: 18.3±7.5→16.3±6.8, p = 0.112; G-II: 17.4±7.5→17.7±7.3, p = 0.791; G-III: 16.9±7.2→17.3±7.3, p = 0.744; G-IV: 17.0±7.2→17.8±6.0, p = 0.435) (Fig 4B). Neither SDS scores (G-I: 32.1±9.1→33.6±9.1, p = 0.233; G-II: 33.4±9.4→33.9±7.9, p = 0.648; G-III: 35.1±9.6→34.3±8.7, p = 0.468; G-IV: 34.2±9.8→34.6±8.3, p = 0.744) (Fig 4C) nor SRS-18 scores (G-I: 19.7±6.3→20.2±5.8, p = 0.606; G-II: 18.6±6.3→19.8±6.0, p = 0.130; G-III: 19.7±6.4→19.5±6.2, p = 0.789; G-IV: 18.7±6.3→20.3±5.7, p = 0.115) (Fig 4D) changed significantly in any group.

## Discussion

There have been several basic and clinical reports of abundant water intake [14,21], tympanic ventilation tubes [15,22] and sleeping in the dark [16,23,24] having possible therapeutic effects on Meniere’s disease. In the present study, one aim was to examine whether these treatments have the feasibility to avoid making a decision of surgery for intractable Meniere’s disease and another was to evaluate which of them is superior. In comparison with a control group, number of vertigo attacks and hearing were significantly better in the water, tube and sleep groups; there were no significant differences between these three groups. Similarly, in comparison with a control group, pAVP concentrations also significantly decreased in the water, tube and sleep groups, again with no significant differences between the three groups. AVP, a stress-related hypothalamic–pituitary releasing hormone, acts via V2R and AQP2 on water metabolism at the level of the renal collecting ducts [25]. Researchers have found that pAVP concentrations are significantly higher in Meniere's patients than in controls without inner ear hydrops [8,17,26]. Takeda has also shown that systemic administration of vasopressin induces bilateral inner ear hydrops and hearing deterioration in guinea pigs [6]. Additionally, long-lasting decreases in pAVP after endolymphatic sac surgery correlate with better surgical results [27]. All these findings suggest that decreased pAVP concentrations after treatment of patients with Meniere’s disease assist control of symptoms.

Possible mechanisms for the therapeutic modalities used in each study group include the following. In G-II: Recently, abundant water intake for Meniere’s disease has been a central therapeutic topic [14]. Although the therapeutic mechanism is unclear, abundant water intake could lead to a decrease in pAVP and may be effective therapy for patients with polycystic kidneys [21]. Conversely, in one animal study, dehydration with associated increased pAVP was harmful to the inner ear [28]. In G-III: Tympanic ventilation tubes helped to reduce inner ear hydrops in an animal hydrops model [15]. Clinically, ventilation tube treatment is reportedly as effective as endolymphatic sac surgery [22]. Tympanic ventilation tubes might correct middle-inner ear pressure, resulting in modification of AVP secretion from the hypothalamic–pituitary system mediated by vestibulo-hypothalamic neural interactions [29]. In G-IV: Generally, patients with Meniere’s disease have some degree of sleep disorder [30]. Therefore, supporting the circadian rhythm of these patients by treating any sleep disorder is an important therapeutic approach. To maintain the hormonal circadian rhythm, including pAVP [16], it is advised to lie in bed in an unlit room for 6–7 hours. Sleeping in darkness may increase pAVP at night and maintain the hormonal circadian rhythm; it is a therapeutic strategy for adults with nocturia [23] and children with enuresis [24]. There are as yet no qualified clinical reports of the effects of sleeping in darkness on vertigo and hearing in Meniere’s disease.

Reportedly, Meniere’s disease is triggered by mental and/or physical stress from participating in demanding activities of daily life and interactions with taxing social environments [3]. However, the proportion of patients experiencing serious stress, especially in greater metropolitan areas, who actually develop Meniere’s disease is relatively small, only 15–50 per 100,000 of the general population [1]. Since temporal bone studies in 1938 first showed that the otopathology in Meniere's disease is inner ear hydrops [4,5], the relationship between stress and this underlying condition has remained unclear. In the 1990s, researchers focused on the role in inner ear function of pAVP, a stress-related anti-diuretic hormone concerned with water metabolism [26]. At the end of the 1990s, its receptor (V2R) and sensitivity were detected in human inner ear endo-organ tissues [31].

Some previous reports have stated that, during remission [8,26] as well as attacks [17], pAVP concentrations in patients with inner ear hydrops are significantly higher than those in patients without this condition. Conversely, other researchers have reported similar pAVP concentrations in patients with unilateral Meniere's disease and healthy controls [32,33]. To clarify these discrepancies, our study group investigated V2R in the inner ear and found a significant negative correlation between pAVP and V2R mRNA expression in the inner ear [8]. Although the mechanism of this negative correlation remains unclear, it may explain why previous studies of pAVP in Meniere's disease have produced a wide and discrepant variety of results. Furthermore, Meniere's patients with high concentrations of both pAVP and V2R in the inner ear characteristically experience clusters of vertigo attacks [8]. Our series of experiments on pAVP and V2R in Meniere’s disease may assist understanding of individual variations in onset and laterality of this disease in the face of the systemic factor of stress.

We would like to emphasize that this study focused on stress hormone vasopressin management rather than stress management. Of course, for Meniere’s patients warm and cordial approaches to reducing mental distress in response to the stresses of daily life are important [3]. However, it is unrealistic for most Meniere’s patients to undertake long-term psychological treatments and/or change their work situations. As shown in Fig 4, vasopressin management using water, tube and/or sleeping in darkness do not require prolonged rest or reduction of stress in daily life. In addition to pAVP, this strategy includes therapeutic targeting of V2R in the inner ear. In an animal study, researchers administered a specific antagonist of V2R systemically to reduce inner ear hydrops [28]. However, in elderly patients, this antagonist could lead to excessive dehydration and subsequent pAVP increases. To prevent such side effects, the epoch-making treatment of local *in vivo* gene therapy through the round window [34] or endolymphatic sac [35] could become feasible in the near future.

Meniere’s disease remains a mysterious inner ear disease. Once patients make a final decision to undergo inner ear surgery, some of them achieve complete freedom from vertigo attacks in advance of the surgery [36]. In addition, even when surgeons fail to identify endolymphatic sacs on the posterior fossa dura during surgery, some patients still achieve such freedom postoperatively [37]. There are various kinds of new drugs and surgeries that sometimes have initial excellent results [38]. In such cases, low pAVP concentrations might also be maintained through those drugs and surgeries as assessed here. Taking together with previous results [6,14,27], we suggest that maintaining low pAVP concentrations could contribute to better treatment results in Meniere’s disease.

Our study has limitations. 1) This clinical study was a randomized-controlled but open-label trial with four arms. In other words, each patient could be aware of his or her own treatment selected out of four. However, during his or her treatment, we checked only his or her treatment rule at every visit: medication compliance in G-I; medication compliance + water intake pattern in G-II; medication compliance + proper placement of ventilation tube in G-III; medication compliance + sleep pattern in darkness in G-IV. We cannot deny that there were some cases with the other treatments in addition to his or her own. 2) During treatment, we made sure who used other drugs, i.e. anti-nausea, sedative and steroids, as seen in Materials and Methods, but not sure how often in using such drugs in each patient and what amounts in each administration. These drug data might be influenced on plasma vasopressin levels. 3) The issue “vasopressin decrease and vertigo suppression: cause or consequence” has been controversial since 1990’s [8,17,26,32,33]. We cannot deny that vasopressin decrease could be one of consequences of vertigo suppression, although we believe vasopressin decrease could be one of causes of vertigo suppression according to previous evidences [6,14,27]. 4) Differences of the number of cases in each group between before and after treatment were 4 in G-I, 5 in G-II, 11 in G-III and 14 in G-IV. These differences could include both cases with treatment failure and complete cure. It was sometimes difficult to understand reasons for dropouts and might be one of limitations in the present study.

To the best of our knowledge, there have never been the similar exploratory studies for treatments of Meniere’s disease. So, we did not do the power analysis to determine the appropriate sample size in each group. However, we would like to believe that the present pilot study of RCT is a good start to find the most feasible strategy. Based on the present pilot data of this RCT, we have already prepared the next trial to determine the most effective combinations for decreasing pAVP, which might enable cure of Meniere’s disease in patients who continue to work hard, even in stressful and demanding social environments.

## Supporting Information

S1 File Checklist for RCT. This file is the filled out CONSORT Checklist for this RCT.
(PDF)

S2 File Changed parts of planning. This file is the changed parts of planning from original to final.
(PDF)

S3 File Approved documents in Japanese. This file shows the original trial protocol of the experiment in this article in Japanese approved by IRB.
(PDF)

S4 File Approved documents in English-1. This file shows the original trial protocol of the experiment in this article in Japanese approved by ClinicalTrialGov.
(JPG)

S5 File Approved documents in English-2. This file shows the original trial protocol of the experiment in this article in Japanese approved by ClinicalTrialGov.
(JPG)

S6 File Approved documents in English-3. This file shows the original trial protocol of the experiment in this article in Japanese approved by ClinicalTrialGov.
(JPG)

S7 File Backgrounds in all cases. This file shows raw data of patients’ backgrounds in all four groups.
(PDF)
