# Supplementary material for: A Two-Year Randomized Trial of Interventions to Decrease Stress Hormone Vasopressin Production in Patients with Meniere’s Disease—A Pilot Study
Source: PLoS One. 2016 Jun 30;11(6):e0158309. doi: 10.1371/journal.pone.0158309 (PMC4928871; doi:10.1371/journal.pone.0158309)
Supplement: S1 File — This file is the filled out CONSORT Checklist for this RCT. (PDF) [file pone.0158309.s001.pdf]

A Checklist for Randomized Controlled Trials (PLOS ONE)

1. Title and Abstract: The study design could not be described so clearly in p.1-2, see a flowchart in **Figure-1**

Introduction

2. Background: reported as **INTRODUCTION** in p.3-4

Methods

3. Participants: see **Patients** in p.4-5
4. Interventions: see **Randomization and treatments** in p.5-6 and a flowchart in **Figure-1**
5. Objectives: reported as **INTRODUCTION** in p.3-4
6. Outcomes: We considered the ratio of the number of improved cases concerned with all the data in the present study, see **Vestibular and Auditory assessments, Laboratory Tests and Questionnaires** and **Statistical analysis** in p.6-8
7. Sample size: We finished this study based on the duration between January, 2010 and December, 2012, see **Patients and Randomization and treatments** in p.4-6, samples in all the four groups shown in **Table-1**

Randomization

8. Sequence generation: see **Randomization and treatments** in p.5-6 and a flowchart in **Figure-1**
9. Allocation concealment: see **Randomization and treatments** in p.5-6 and a flowchart in **Figure-1**
10. Implementation: see **Randomization and treatments** in p.5-6 and a flowchart in **Figure-1**
11. Blinding/Masking: Blinding was performed only for patients (not for doctors) in the present study, see **Randomization and treatments** in p.5-6 and a flowchart in **Figure-1**
12. Statistical methods: see **Statistical analysis** in p.8

Results

13. participant flow: see **Randomization and treatments** in p.5-6 and a flowchart in **Figure-1**
14. Recruitment: see **Randomization and treatments** in p.5-6 and a flowchart in **Figure-1**
15. Baseline data: reported as **INTRODUCTION** in p.3-4
16. Number analyzed: described in **RESULTS** in p.8-9 and in **Table-1**

17. Outcomes and estimation: described in **RESULTS** in p.8-9 and in **Table-2**
18. Ancillary analyses: see **Statistical analysis** in p.8 and **ACKNOWLEDGEMENTS** in p.14
19. Adverse events: effects and side effects were described as a flowchart in **Figure-1** and % graphs in **Figures 2,3**

Comment

20. Interpretation: Interpretation (the 1st paragraph in p.9-10) and limitation (the 6th paragraph in p.12-13) in the present study were described in **DISCUSSION**
21. Generalizability: The present findings encourage us to think further ideal treatments beyond this paper and that was described in DISCUSSION (the 5th paragraph in p.12)
22. Overall evidence: described in DISCUSSION (1ine.13-15 at the end of the 1st paragraph in p.10)
