# Supplementary material for: A Two-Year Randomized Trial of Interventions to Decrease Stress Hormone Vasopressin Production in Patients with Meniere’s Disease—A Pilot Study
Source: PLoS One. 2016 Jun 30;11(6):e0158309. doi: 10.1371/journal.pone.0158309 (PMC4928871; doi:10.1371/journal.pone.0158309)
Supplement: S2 File — This file is the changed parts of planning from original to final. (PDF) [file pone.0158309.s002.pdf]

This supplement contains the following items:

1. Protocol: original protocol, final protocol, summary of changes

2. Statistics: original statistical analysis plan, final statistical analysis plan, summary of changes

1. Protocol:

Original protocol

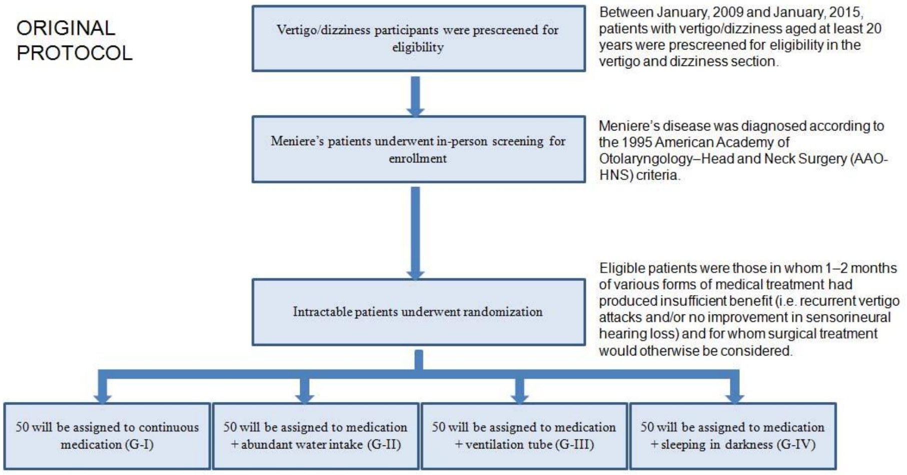

Group-I (G-I) continued to receive traditional oral medication.
Group-II (G-II) received both medication and abundant water intake at least 2.0 litters per day with a self-check diary of water volume/day.
Group-III (G-III) received medication and had ventilation tubes inserted through an incision in their tympanic membranes under local anesthesia to relieve inner ear hydrops.
Group-IV (G-IV) received both medication and advice to sleep regularly in darkness to maintain the hormonal circadian rhythm, defined as lying in bed in an unlit room with a self-check diary using an illuminometer.

Final protocol

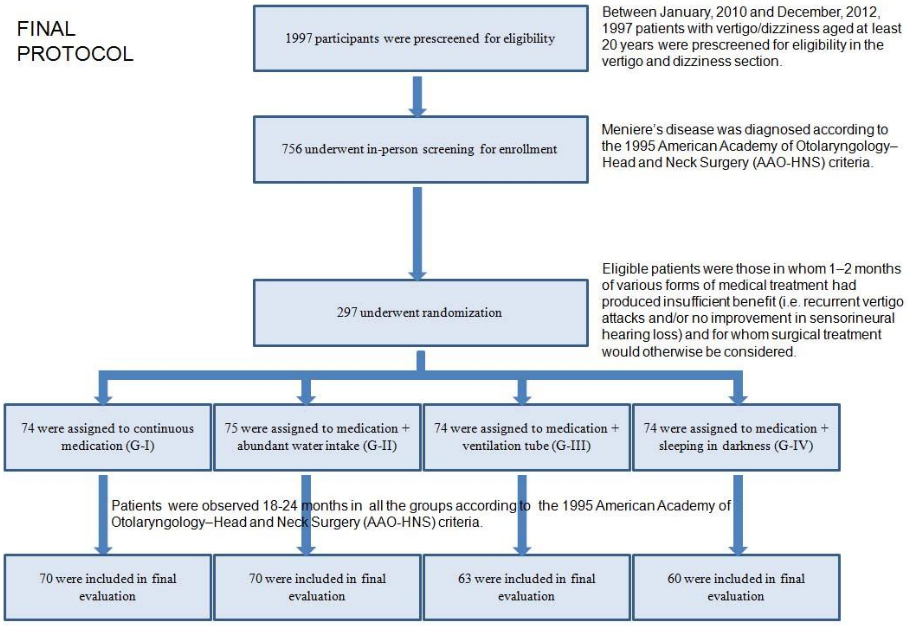

Group-I (G-I) continued to receive traditional oral medication.
Group-II (G-II) received both medication and abundant water intake: 35 mL/kg/day as specified by a previous study with a self-check diary of water volume/day.
Group-III (G-III) received medication and had ventilation tubes inserted through an incision in their tympanic membranes under local anesthesia to relieve inner ear hydrops.
Group-IV (G-IV) received both medication and advice to sleep in darkness to maintain the hormonal circadian rhythm, defined as lying in bed in an unlit room less than 1.0 lux for 6–7 hours per night with a self-check diary using an illuminometer.

Summary of changes

1) A principal investigator, Tadashi Kitahara, moved from an Associate Professor of Osaka University to a Professor & chairman of Nara Medical University.

   REASONS: Just a promotion matter in May 1st, 2014.

2) Periods of patients' enrollment changed from January, 2015 to December, 2012 and those of clinical observation changed from January, 2020 to December, 2014.

   REASONS: We could enroll cases more promptly than we expected.

3) Numbers of patients changed from 200 (50 in each group) to 263 (G-I 70, G-II 70, G-III 63, G-IV 60).

   REASONS: We could enroll more cases than we expected.

4) More detailed description of the condition of Medication + Water intake Group (G-II) was made as 35 mL/kg/day in the final protocol.

   REASONS: We clinically did it from the beginning of this study but did not describe in the start protocol.

5) More detailed description of the condition of Medication + Regular sleep Group (G-IV) was made as unlit room less than 1.0 lux for 6-7 hours in the final protocol.

   REASONS: We clinically did it from the beginning of this study but did not describe in the start protocol.

6) Nothing else was changed between the original and final.

2. Statistics:

Original statistical analysis plan

As seen in our manuscript, all treatment results were expressed as ratios of the number

of cases and assessed statistically by SPSS version 16.0 (Chicago, IL). For post-treatment results, the χ^2 test (for vertigo) and Mann-Whitney U-test (for hearing) were used to compare various pairs of the four groups, G-I, G-II, G-III, and G-IV. Student's paired *t*-test and repeated measures ANOVA were used to examine the statistical significance of changes in laboratory data and questionnaire points after each treatment. All reported p-values were two-sided and those under 0.05 were considered significant.

Final statistical analysis plan

Numbers of patients changed from 200 (50 in each group) to 263 (G-I 70, G-II 70, G-III 63, G-IV 60). The statistical analysis was performed in the same way as above.

Summary of changes

Nothing in the way of statistical analysis was changed between the original and final.
