# Supplementary material for: A Two-Year Randomized Trial of Interventions to Decrease Stress Hormone Vasopressin Production in Patients with Meniere’s Disease—A Pilot Study
Source: PLoS One. 2016 Jun 30;11(6):e0158309. doi: 10.1371/journal.pone.0158309 (PMC4928871; doi:10.1371/journal.pone.0158309)
Supplement: S3 File — This file shows the original trial protocol of the experiment in this article in Japanese approved by IRB. (PDF) [file pone.0158309.s003.pdf]

様式２

受付番号	2263
区　　分	■新規　□変更　□その他

西暦２０１０年０４月１６日

研 究 計 画 書

１．課　題　名	メニエール病患者のストレス・マネジメント治療
公開用課題名	メニエール病患者のストレス・マネジメント治療
２．研究の目的及び実施計画の概要（公開）	現在までにメニエール病に対して１.水分大量摂取、２.鼓膜ﾁｭｰﾌﾞ留置、３.良質の睡眠の３案が、症例によってある程度効果があると報告されている。今回の研究は診療範囲内の研究で、当該期間に受診したメニエール病確実例に対して、従来の基本的内服治療である浸透圧利尿薬、循環改善薬、抗めまい薬を投与するとともに、無作為にストレス・マネジメント(水分大量摂取、鼓膜ﾁｭｰﾌﾞ留置、良質の睡眠、健胃薬)を追加する研究である。治療開始後、定期的に１年間、めまい発作頻度および聴力レベルの推移を観察し、効果判定していく。試験デザインは非盲検無作為化並行群試験である。
３．研究責任者（公開）<br>氏名・所属・職	北原　糺・耳鼻咽喉科・頭頸部外科・部長
４．研究実施予定期間（公開）	承認後４年間

５．研究分担者<br>氏名・所属・職	梶川　泰　・耳鼻咽喉科・頭頸部外科・副部長<br>福嶋　宗久・耳鼻咽喉科・頭頸部外科・医員<br>古川　雅史・耳鼻咽喉科・頭頸部外科・医員<br>大畠　和也・耳鼻咽喉科・頭頸部外科・医員
６．研究協力者<br>氏名・所属・職	とくになし
７．連絡先	氏名：　北原　糺　所属：　耳鼻咽喉科・頭頸部外科　職：　部長<br>内線番号：　　PHS 番号：<br>E-mail:　tkitahara@ent.med.osaka-u.ac.jp

８．研究の実施場所
大阪労災病院　耳鼻咽喉科・頭頸部外科

９．研究の背景
メニエール病は原因不明の疾患であるが、以前からストレスにより発症することが報告されている。ここ 20 年の基礎および臨床研究により、メニエール病患者の血中抗利尿ホルモンおよび内耳抗利尿ホルモン受容体が対照患者に対して有意に上昇しており、その両者の上昇が内耳に悪影響を及ぼすことがわかった。一方、水分大量摂取、鼓膜ﾁｭｰﾌﾞ、良質の睡眠によりそれぞれ症例によってはある程度の効果があるとの報告が散見されているが、それらの治効機序は不明である。本研究は、精神的、肉体的ストレスにより視床下部―下垂体で分泌が増加するストレス・ホルモンである抗利尿ホルモンを、これらのストレス・マネジメントによって低下させることができるか、さらに臨床症状を軽減させることができるかを検討するための研究である。

１０．研究計画
１．目　的
本研究は、精神的、肉体的ストレスにより視床下部下垂体で分泌が増加するストレス・ホルモンである抗利尿ホルモンを、何らかの方法で低下させる治療を考案するための研究である。

２．対　象
受診したメニエール病確実例患者。確定診断は厚生労働省の診断基準による。症例は毎年 50 例弱であるから、4 年間で 200 症例とする。とりあえず探索的に、最初の１年目 50 例で解析してみる。なおメニエール病は未成年に発症することは稀であるから、対象は 20 歳以上とする。
なお、４群のいずれかに割り当てられた際にその治療法を拒否した患者は、本研究対象から除外して希望の治療を行う。

３．方　法
現在までにメニエール病に対する効果が報告されている、１．水分大量摂取(１日 2.0 ﾘｯﾄﾙ以上毎日)２．鼓膜ﾁｭｰﾌﾞ留置(責任耳側に外来時に施行)、３．良質の睡眠(暗所睡眠で睡眠時間・起床時間床を記録)、の３案をメニエール病確実例に勧める。申請期間内に受診したメニエール病確実例に対して、従来の基本的内服治療である浸透圧利尿薬、循環改善薬、抗めまい薬の投与を続行するとともに、上記の３案に健胃薬の内服を追加する群を含めた４群に分ける。同意は同意書を用いて各症例に対して行う。患者登録と割付は、大阪労災病院における耳鼻咽喉科・頭頸部外科データセンター(管理者：山下智子)において行う。検査項目、調査項目は臨床症状(めまい発作頻度)、血液(ストレス・ホルモン)、神経耳科学的所見(聴力レベル)の推移を観察する。なお、初診時腎機能異常を呈する患者はグループ１から除外する(末血、生化)。

割付方法は置換ブロック法で割付
１）データセンター（管理者：山下智子）において、無作為割り付けの方法として、予め置換ブロック法（並べかえブロック法）にて割り付け表を作成しておく。（鍵のかかる引出しなどに保管し、他の人がみることができない。）
２）治療を行う医師は、患者さんの同意を得た後、データセンターに電話で連絡し、データセンター管理者は、患者を登録する。
３）登録完了後、割り付け結果（A または B など）を、治療を行う医師に、電話で連絡する。
４）研究終了時にデータセンターでの割付情報と、治療を行う医師の割付情報とを照合し、一致していることを確認する。

４．評価方法
臨床症状、血液、神経耳科学的所見の推移を観察する。臨床症状はめまい、うつ、ストレスのそれぞれに対する質問表を用い、血液所見はコルチゾルやバゾプレッシン等のストレス・ホル

モン、神経耳科学的所見は純音聴力検査を、初診時、１ヶ月、２ヶ月、６ヶ月、１２ヶ月と決められた時期に検索する。また、同時にストレス・マネジメント達成度も確認する。達成度は自己申告制とし、50%未満と見なされる場合は本研究対象から除外する。

５．期待される成果
対照となる従来の内服治療に加え、水分、鼓膜ﾁｭｰﾌﾞ、睡眠などの比較的容易に実現可能である因子が、メニエール病患者のストレス・ホルモンを減少させ、さらには前庭、聴覚系に良い影響を与えるかを検索する。得られた結果によって、メニエール病患者に従来の内服治療に加え、より効果的な治療法を提供できると考える。

１１．医学的意義
メニエール病患者の中には、従来の内服治療でめまいおよび難聴の軽快しない難治性症例が、約20%程度存在する。難治性症例に対しては、全身麻酔下での内リンパ嚢開放術、局所麻酔下でのゲンタマイシン中耳腔投与など、侵襲的な治療法しかない。したがって本研究により、日常的に実行可能な非観血的ストレス・マネジメント治療が確立されることによる医学的意義は大きい。

１２．社会的意義
先述したごとく、メニエール病は原因不明の疾患であるが、以前からストレスにより発症することが報告されている。従来、耳鼻咽喉科医はメニエール病患者に対して「ストレスのないゆったりした生活を送りなさい」という通り一遍の生活指導をすることが多かった。しかしながら昨今の厳しい社会情勢の中で、そのような生活指導は非現実的であると言わざるを得ない。したがって本研究により、日常的に実行可能な非観血的ストレス・マネジメント治療が確立されることによる社会的意義は大きい。

１３．研究対象者の安全に関する問題点とその対策
本研究は、受診したメニエール病確実例に対して、必要事項の問診、質問表、血液検査、聴力検査等を行うのみである。急性増悪時には点滴加療を行い、場合によっては手術加療に移行することも準備しておく。したがって、本研究期間内に受診する患者が、それ以外の期間に受診する患者に比べて、不可逆的な不利益にさらされることはない。

１４．その他の研究に伴う問題点とその対策
とくになし。

１５．研究終了後の対応　　（該当するものはすべてに■として下さい）
■研究成果は、研究対象者を特定できないようにした上で、学会や学術雑誌等で公表する。

□研究対象者のデータは完全に廃棄する。（電子データについては完全に消去する）

■研究対象者のデータは個人情報を厳重に管理（匿名化など）した上で保存する。
（保存期間　：　５年間　）
（保存責任者：　北原　糺　）

■研究対象者の検体（血液等のヒト由来の試料）は、定期的血液検査時ごとに必要量採取し使い切る。

□研究対象者の検体（血液等の検査のためのヒト由来の試料）は研究対象者の同意のもと保存する。
（保存する理由：　　　　　　　　　　　　　　　　　　）
（保存する場所：　　　　　　　　　　　　　　　　　　）
⇒この場合は包括同意説明文書・包括同意書・撤回書が必要です。

□その他
（具体的に：　　　　　　　　　　　　　　　　　　　　　　　　　　　　　　）

１６．**プライバシーの確保に関する対策**　①～③で該当するものを■として下さい
■①「連結可能匿名化」を行い、個人情報を保護する。
（研究対象者のデータや検体から氏名等の個人情報を削り、代わりに新しく符号又は番号をつけて匿名化を行う。研究対象者とこの符号（番号）を結びつける対応表は外部に漏れないように厳重に保管する）

（対応表の管理方法）該当するものを■として下さい
■ネットワークから切り離されたコンピューターを使用して、外部記憶媒体（USB など）に記録され、それは鍵をかけて厳重に保管する。
■筆記による紙媒体として、鍵をかけて厳重に保管する。
□その他（具体的に：　　　　　　　　　　　　　　　　　　　　　　　　　　　　）

□②「連結不可能匿名化」を行い、個人情報を保護する。
（研究対象者のデータや検体から氏名等の個人情報を削り、代わりに新しく符号又は番号をつけて匿名化を行う。研究対象者とこの符号（番号）を結びつける対応表は作成しない。）

□③その他
（具体的に：　　　　　　　　　　　　　　　　　　　　　　　　　　　　　　　）

１７．**個人情報管理者**
所属：　耳鼻咽喉科・頭頸部外科　職名：　部長　氏名：　北原　糺

１８．**インフォームド・コンセントの手続き**
インフォームド・コンセントの有無について

■研究対象者から、文書によるインフォームド・コンセントを得て研究を行う。
（19 へ）
□代諾者等から、文書によるインフォームド・コンセントを得て研究を行う。（1～3 記入後、19 へ）
＊「同意書・同意撤回書作成についての留意点」を参照して下さい

１．代諾者等の選定方針

２．研究対象者の参加が研究を実施するにあたり必要不可欠な理由

３．本研究の重要性

□インフォームド・コンセントを省略して研究を行う。
（→20 へ）

□その他（具体的に：　　　　　　　　　　　　　　　　　　　　　　　　　　　　）

１９．**説明文書に記載したインフォームド・コンセントの内容**

■①研究への参加は任意であること、同意しない場合も不利益を受けないこと
■②いつでも同意を撤回しても何ら不利益を受けないこと
■③研究の目的、意義及び研究期間（研究全体の期間）
■④研究の方法（研究対象者として選定された理由 等）
■⑤他の治療方法の有無
■⑥研究により期待される結果及び起こりうる危険性・不便
■⑦個人情報の取扱い
■⑧研究終了後の対応・研究成果の公表について
■⑨試料（資料）の保存及び使用方法並び保存期間（研究終了後の試料（資料）取扱いについて）
■⑩費用負担に関すること
■⑪健康被害が発生した場合の対処と補償の有無
□⑫当該研究に係る資金源
□⑬起こりうる利害の衝突及び研究者等と関連組織との関わり
■⑭研究の開示
□⑮当研究の結果を他の機関へ提供する可能性
■⑯知的財産権等の帰属
□⑰共同研究の場合のその内容
■⑱研究に関する問い合わせ先
■⑲研究機関名・研究者等の氏名・職名・連絡先
□⑳【被験者からインフォームド・コンセントを得ることが困難な場合】研究の重要性、被験者の参加が研究実施に当たり必要不可欠な理由

２０．インフォームド・コンセントを省略して研究を行う場合以下の措置が必要

（不明な点は臨床試験部に問い合わせて下さい。）

□1) 研究開始前に研究の目的を含む研究の実施についての以下の情報を当科ホームページ（アドレスを記載して下さい）上で公開する。

①対象
②研究機関名
③目的
④方法
⑤意義
⑥個人情報の扱い
⑦問い合わせ先

□2) 研究開始前に研究の目的を含む研究の実施についての以下の情報を当科ホームページ（アドレスを記載して下さい）上で公開し、研究対象者が対象者になることを拒否できるようにする。

①対象
②研究機関名
③目的
④方法
⑤意義
⑥個人情報の扱い
⑦問い合わせ先
⑧研究対象者に研究への参加を拒否する権利を与える方法

□3）匿名化を行う。（連結不可能匿名化又は連結可能匿名化であって対応表を提供しない場合）
□4）情報を公開しない。（理由：　　　　　　　　　　　　　　　　　　　　　　　　　　　）
□5）その他（具体的に：　　　　　　　　　　　　　　　　　　　　　　　　　　）

２１．研究に伴い研究対象者に生じた健康被害の補償のための保険等必要な措置

■①補償なし。

□②補償あり。
（具体的内容：　　　　　　　　　　　　　　　　　　　　　　　　　　　）

□③その他
（具体的内容：　　　　　　　　　　　　　　　　　　　　　　　　　　　）

２２．本研究の資金源、実施により起こりうる利害の衝突

とくになし。

２３．本研究課題についての他機関等による審査状況

本研究は大阪労災病院のみで行うので該当しない。

２４．その他参考となる事項

とくになし。
