# Supplementary figures and images for: A Two-Year Randomized Trial of Interventions to Decrease Stress Hormone Vasopressin Production in Patients with Meniere’s Disease—A Pilot Study
Source: PLoS One. 2016 Jun 30;11(6):e0158309. doi: 10.1371/journal.pone.0158309 (PMC4928871; doi:10.1371/journal.pone.0158309)

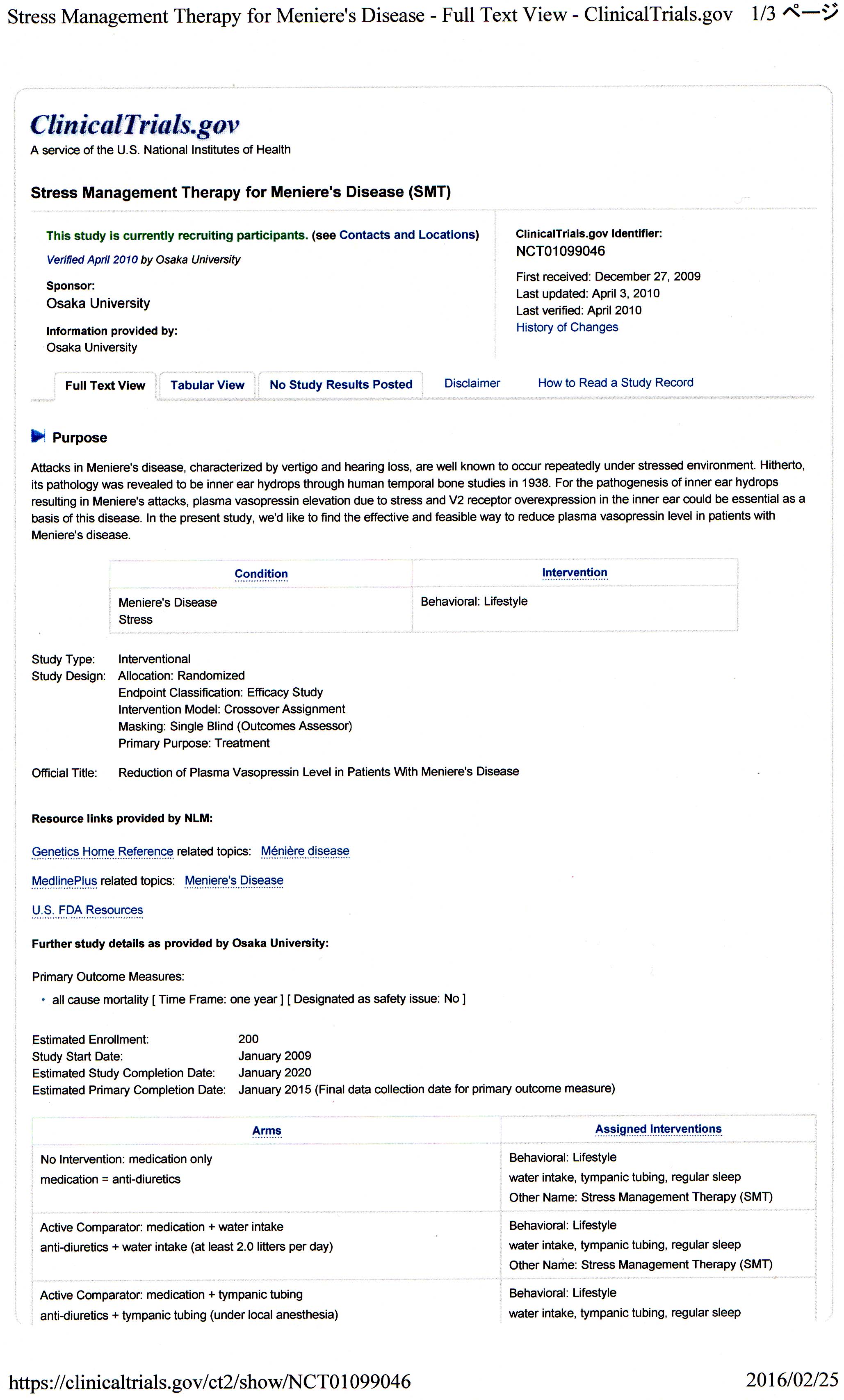

Supplement: S4 File — This file shows the original trial protocol of the experiment in this article in Japanese approved by ClinicalTrialGov. (JPG) [file pone.0158309.s004.jpg]

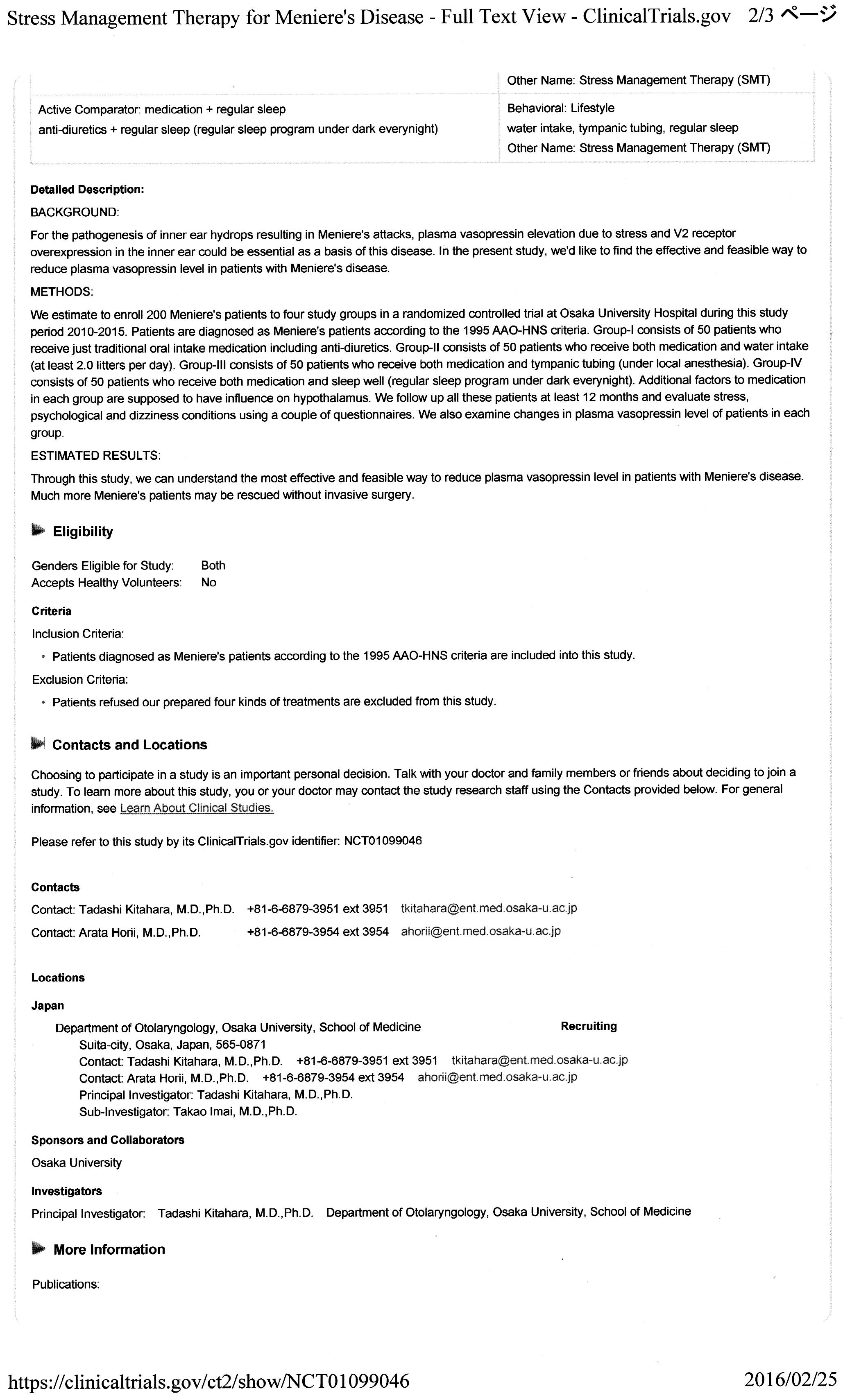

Supplement: S5 File — This file shows the original trial protocol of the experiment in this article in Japanese approved by ClinicalTrialGov. (JPG) [file pone.0158309.s005.jpg]

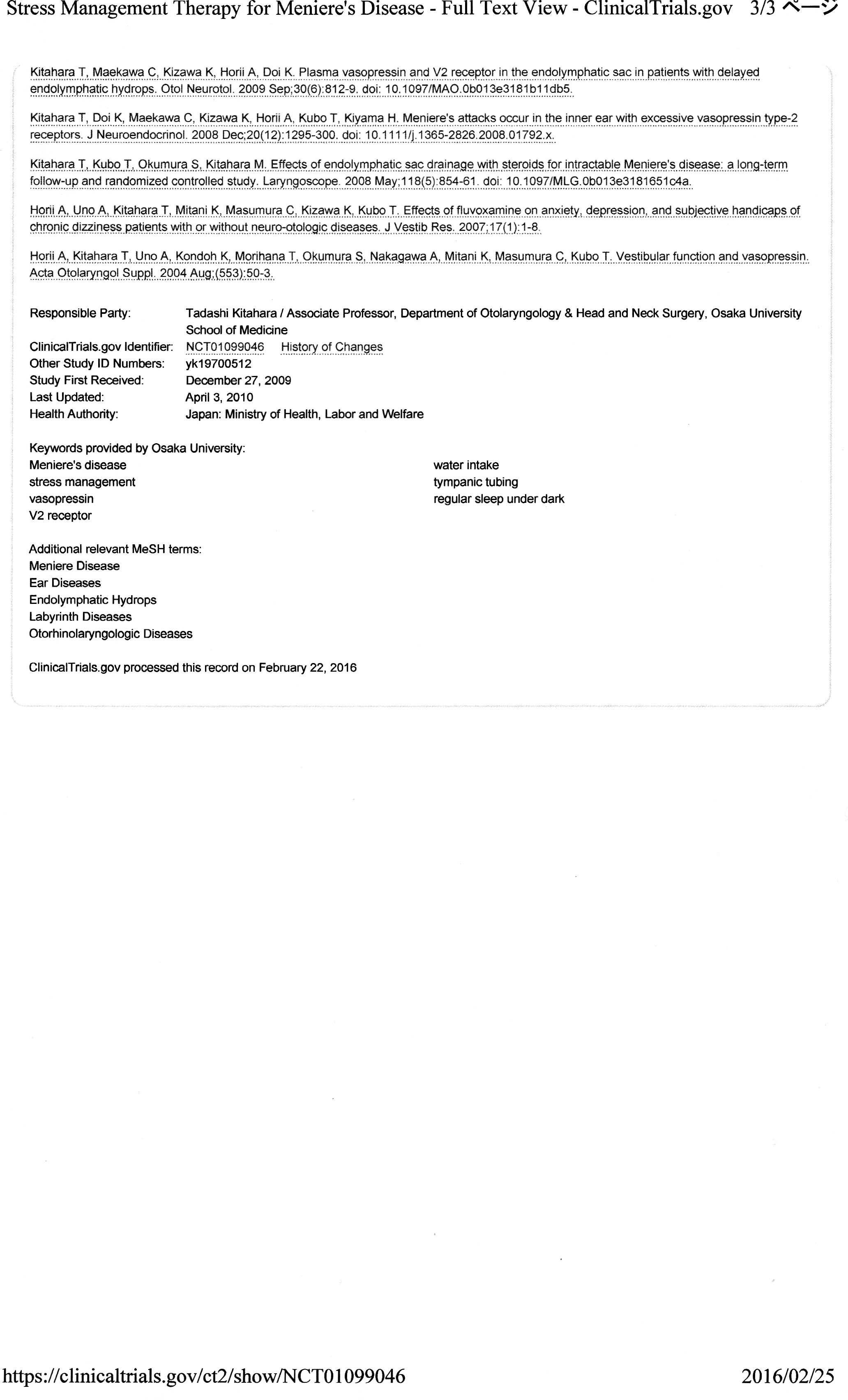

Supplement: S6 File — This file shows the original trial protocol of the experiment in this article in Japanese approved by ClinicalTrialGov. (JPG) [file pone.0158309.s006.jpg]
