# Supplementary material for: A Two-Year Randomized Trial of Interventions to Decrease Stress Hormone Vasopressin Production in Patients with Meniere’s Disease—A Pilot Study
Source: PLoS One. 2016 Jun 30;11(6):e0158309. doi: 10.1371/journal.pone.0158309 (PMC4928871; doi:10.1371/journal.pone.0158309)
Supplement: S7 File — This file shows raw data of patients’ backgrounds in all four groups. (PDF) [file pone.0158309.s007.pdf]

	sex	age	lat	dur	avp	crt	sds	srs	vf	hl	
101	1	30	1	24	2	10	24	22	0.5	35.8	G-1
102	1	31	1	10	2	25	30	16	0.5	35.5	G-1
103	0	40	1	15	1.3	22	40	22	0.5	46	G-1
104	0	31	0	10	1.8	16	22	14	1.7	70.5	G-1
105	0	45	1	24	3.4	8	40	23	2	48	G-1
106	1	56	0	32	2.3	10	24	17	2.7	40.2	G-1
107	0	54	0	6	1.9	24	40	12	0.5	33.6	G-1
108	0	47	1	12	1.4	25	25	15	4	42.3	G-1
109	1	68	1	14	6.7	18	42	11	1.7	65.5	G-1
110	0	21	0	9	2.9	10	24	13	2.5	38	G-1
111	0	62	1	10	11	14	26	31	1.7	33	G-1
112	1	78	0	12	4.4	22	40	22	1	62.6	G-1
113	0	23	0	9	4.2	16	25	20	1	38.8	G-1
114	0	38	1	20	2.1	25	40	14	2.5	40.3	G-1
115	0	71	0	12	4.6	30	46	22	3	34	G-1
116	0	68	1	30	13	25	22	12	0.5	28.8	G-1
117	0	61	1	35	6.3	30	24	13	0.5	74.5	G-1
118	1	27	0	3	3.4	12	40	23	1	35	G-1
119	0	55	1	36	3.8	6	46	36	4	35	G-1
120	0	72	0	45	4.1	8	24	24	1.5	45.4	G-1
121	0	81	1	70	4.2	14	50	12	2.3	33.5	G-1
122	1	35	0	36	1.5	19	44	21	1.7	42.8	G-1
123	0	35	0	10	3.7	26	40	24	1.7	60	G-1
124	0	30	0	18	6.9	30	25	22	2	54.6	G-1
125	0	67	0	120	2.4	12	50	22	3.3	28	G-1
126	1	39	0	24	2	14	24	25	0.5	70	G-1
127	0	35	1	48	4.5	15	26	31	3.3	44.9	G-1
128	0	44	0	66	11	10	40	16	2.7	43.5	G-1
129	0	63	1	45	4.7	10	24	13	3	51.5	G-1
130	0	44	1	10	1.5	33	28	31	2	59	G-1
131	1	50	0	20	1.7	30	28	15	1.5	53.4	G-1
132	0	41	1	36	3.9	25	22	16	2	62.6	G-1
133	0	36	1b	36	4.9	15	42	22	0.5	38.8	G-1
134	0	32	0b	48	3.9	25	46	14	0.5	40.3	G-1
135	1	69	0b	27	0.6	11	52	34	3.5	40.5	G-1
136	0	73	1	33	1.4	10	28	31	3.3	28.8	G-1
137	0	30	1	6	3.1	19	26	18	1	43	G-1
138	0	27	0	14	0.5	22	33	15	1	48	G-1
139	0	40	1	25	2.9	26	24	25	0.5	55	G-1
140	1	48	1	12	0.8	10	30	14	1	28	G-1
141	0	56	0	6	1.7	26	22	25	0.5	33.5	G-1
142	1	75	0	44	5.6	15	26	30	1.7	28	G-1
143	1	69	1	35	1.7	25	32	30	2.3	60	G-1
144	0	77	0	60	1.5	16	44	15	1.7	54.6	G-1

	sex	age	lat	dur	avp	crt	sds	srs	vf	hl	
201	1	74	1	48	0.8	25	44	12	3	43.8	G-2
202	0	43	0	36	1.7	18	32	15	0.5	44	G-2
203	0	48	0	36	2.7	10	24	20	0.5	50	G-2
204	0	43	0	48	6.8	14	28	30	1	48.4	G-2
205	0	54	1	27	2.2	22	46	20	4	38.5	G-2
206	0	77	0b	33	0.8	16	33	12	1.5	53.1	G-2
207	1	67	0	6	2	25	24	20	2.3	40.4	G-2
208	0	41	0	14	1.5	30	30	15	1.7	33.4	G-2
209	0	62	0	25	1.4	25	22	21	1.5	65	G-2
210	0	47	0	12	1.8	15	26	12	1.3	58.8	G-2
211	1	41	1	4	4.4	12	22	13	3	46	G-2
212	0	62	0	45	2.6	6	44	20	0.5	31.3	G-2
213	0	66	0	100	11	8	40	10	3.3	68	G-2
214	0	47	0	8	1.2	14	25	20	2.7	55.7	G-2
215	1	24	1	12	4.2	19	24	23	3	48.8	G-2
216	0	49	1b	30	1.4	26	24	11	2	60	G-2
217	1	42	1	22	1.7	30	26	12	1.5	43.8	G-2
218	0	31	0b	20	4.2	12	46	20	2	73.8	G-2
219	1	56	0b	48	1.6	14	32	23	0.5	50	G-2
220	1	36	1	3	2.8	15	48	12	2	37.5	G-2
221	0	57	0b	30	7.5	10	24	25	1.5	62.5	G-2
222	0	33	0	36	4.7	10	42	31	2	23.8	G-2
223	1	66	0	96	2.5	33	48	20	0.5	61.3	G-2
224	0	28	1b	22	4.1	30	44	24	1	68	G-2
225	1	36	0	27	2.1	25	24	25	1	22.5	G-2
226	1	53	1	98	9.5	15	45	22	0.5	77.5	G-2
227	1	58	1b	56	2.1	10	22	36	1.7	37.5	G-2
228	0	24	0	35	1.6	11	32	20	2	63.8	G-2
229	0	51	0	44	0.7	10	25	12	2.7	40	G-2
230	1	66	1	40	1.3	19	24	14	0.5	48	G-2
231	0	32	0	12	1.1	22	30	19	3.3	75	G-2
232	1	51	1	66	3.2	26	32	30	2.3	28.2	G-2
233	0	42	1b	40	1.5	10	45	31	2	33.6	G-2
234	0	47	0	6	3.5	14	28	23	0.5	25	G-2
235	0	62	1b	12	3	10	38	26	1.7	63.8	G-2
236	1	50	1	33	1	5	22	14	0.5	38.8	G-2
237	0	56	1	20	12	28	24	13	1.3	40.3	G-2
238	1	40	1	6	2.9	5	33	16	1	40.5	G-2
239	0	44	0b	28	4.8	25	50	22	2.5	28.8	G-2
240	0	44	1	36	1.4	22	28	15	0.5	46	G-2
241	0	24	0b	15	3.1	28	50	11	0.5	68.6	G-2
242	0	60	0b	70	3.4	12	36	13	1	55	G-2
243	1	58	1	12	3.9	10	24	16	1	45.4	G-2
244	1	38	1	15	1.6	5	28	22	0.5	33.5	G-2

	sex	age	lat	dur	avp	crt	sds	srs	vf	hl	
301	0	49	0	42	4.1	30	38	21	2	30	G-3
302	1	62	0	60	2.1	26	22	31	1.5	71.3	G-3
303	0	32	1	48	0.5	15	49	12	2	46	G-3
304	0	52	0b	44	2.1	25	22	15	0.5	70.5	G-3
305	0	60	0	15	1.6	16	24	16	2.5	32.3	G-3
306	1	58	0	22	0.7	23	28	23	3.5	40.2	G-3
307	1	36	0	35	5.2	26	29	24	3.3	33.6	G-3
308	0	70	1b	40	6.6	20	34	18	4	42.3	G-3
309	1	73	0b	36	3.2	11	52	31	4	65.5	G-3
310	0	57	0	35	1.5	10	44	12	0.5	69.4	G-3
311	0	68	0	44	3.5	5	24	16	1	53.4	G-3
312	0	58	1	40	3	18	25	25	0.5	62.6	G-3
313	0	58	1b	12	1	20	33	24	1.7	38.8	G-3
314	0	53	1	56	3.4	6	33	25	2.3	40.3	G-3
315	1	71	0	40	2.3	19	40	23	4	30	G-3
316	0	42	0b	6	1.9	25	48	35	0.5	28.8	G-3
317	0	73	1b	12	1.4	22	50	14	0.5	45	G-3
318	0	32	1	33	6.7	12	26	16	1	56.3	G-3
319	0	41	0	20	2.9	15	24	17	4	62	G-3
320	1	40	1b	40	6.8	8	28	18	1.5	47.5	G-3
321	0	75	0	9	4.4	12	32	22	2.3	36	G-3
322	0	41	0	20	11	15	34	13	1.7	61	G-3
323	1	58	0	12	2.1	20	38	14	1.5	28	G-3
324	1	72	0	30	4.6	8	44	12	1.3	55	G-3
325	0	49	0	118	9.2	14	25	23	3	73.8	G-3
326	1	59	0	45	6.3	19	28	11	0.5	36.3	G-3
327	0	58	1	78	9.6	26	36	21	3.3	76.3	G-3
328	0	62	1	36	3.8	30	28	12	2.7	51.3	G-3
329	1	52	1	36	4.1	12	36	12	3	55	G-3
330	0	56	1	48	4.2	14	48	15	2	48.8	G-3
331	1	36	0b	27	1.5	15	52	24	1.5	43.8	G-3
332	0	26	0b	20	3.7	10	24	31	2	66.3	G-3
333	1	62	0b	44	6.9	22	40	34	0.5	36.3	G-3
334	0	68	1	44	2.4	25	46	26	1	41.3	G-3
335	1	45	0	48	2	28	24	19	1	62.5	G-3
336	0	46	1	66	4.5	15	50	28	0.5	52.5	G-3
337	1	59	1	80	2.8	15	44	16	1.7	55.6	G-3
338	0	30	1	50	4.7	20	50	12	2	56.3	G-3
339	0	68	1	36	5.7	10	45	24	2.7	58.8	G-3
340	1	45	1	6	1.7	12	25	25	0.5	36	G-3
341	0	23	1	40	3.9	6	24	13	4	65	G-3
342	0	38	1	60	4.9	18	26	12	1.7	70	G-3
343	1	36	0	30	3.9	22	40	14	2.5	55	G-3
344	0	63	1	24	6.8	12	24	16	1.7	35	G-3

145	1	28	1	3	3	23	22	16	3.3	48	G-1
146	0	74	1	48	1.3	26	25	17	2.5	70	G-1
147	0	62	0	66	2.6	20	24	18	2.5	46	G-1
148	1	59	1b	96	15	11	48	22	0.5	70.5	G-1
149	0	73	0	50	1.1	10	38	13	1.7	67.5	G-1
150	0	42	1	36	1.8	5	22	14	0.5	40.2	G-1
151	1	56	0	6	2	18	24	18	1.3	33.6	G-1
152	1	65	1	40	0.4	20	33	14	1	42.3	G-1
153	0	67	1	60	0.3	6	30	18	2.5	65.5	G-1
154	1	46	0	30	3.8	19	48	24	0.5	69.4	G-1
155	1	46	0	24	0.9	25	24	31	0.5	43	G-1
156	1	58	0	48	1.4	22	46	12	0.5	32.5	G-1
157	0	68	1	68	15	12	24	20	1	66.3	G-1
158	0	44	1	3	1	15	28	23	1	48.4	G-1
159	0	33	0b	24	5.9	8	32	12	0.5	38.5	G-1
160	0	25	1	3	0.8	18	34	25	1.7	45	G-1
161	0	48	0	35	2.9	15	38	12	2	40.4	G-1
162	0	48	1	44	4.8	10	24	13	0.5	33.4	G-1
163	1	51	1b	40	1.4	6	25	25	0.5	65	G-1
164	0	28	1	12	3.1	25	28	19	4	58.8	G-1
165	1	65	1	56	3.4	30	36	13	1.7	66	G-1
166	1	32	0	40	3.9	24	25	20	2.5	31.3	G-1
167	1	40	1	6	1.6	20	22	12	1.7	68	G-1
168	0	53	1	12	9.6	18	32	22	1	55.7	G-1
169	1	69	1b	33	0.7	33	42	22	1	48.8	G-1
170	0	33	1	20	1.2	28	22	21	0.5	30	G-1
70	25	49.7	39	30.3	3.5	18.3	32	20	1.6	47.3	
	45	16.5	31	22.7	3.2	7.5	9.1	6.3	1	13.4	

245	0	36	1	24	6.8	25	32	10	0.5	42.8	G-2
246	0	20	1	3	0.7	10	34	14	0.5	47	G-2
247	0	32	1	30	11	15	38	12	2	55.7	G-2
248	1	75	1b	48	1.7	18	44	24	2.7	48.8	G-2
249	1	61	1b	38	6.8	11	25	18	0.5	60	G-2
250	0	25	1	9	2.2	22	28	31	4	43.8	G-2
251	0	44	0	3	0.8	8	36	12	1.7	73.8	G-2
252	0	42	0	25	2	10	28	16	2.5	50	G-2
253	0	36	1	24	1.5	14	36	15	1.7	37.5	G-2
254	1	30	1b	20	1.4	15	48	24	1	62.5	G-2
255	0	60	0	72	1.8	22	22	10	1	23.8	G-2
256	0	51	0b	28	4.4	20	24	23	2.5	61.3	G-2
257	0	36	1	24	2.6	18	40	31	3	48	G-2
258	1	39	0	48	1	28	46	14	0.5	22.5	G-2
259	0	40	0	66	1.2	32	24	18	0.5	77.5	G-2
260	1	34	1	45	4.2	22	50	27	1	37.5	G-2
261	0	64	1	10	1.4	15	24	18	4	63.8	G-2
262	1	60	1	20	4	18	25	12	1.5	40	G-2
263	1	40	1	36	4.2	25	50	23	2.3	66.7	G-2
264	1	41	1	36	1.6	11	24	14	1.7	75	G-2
265	0	28	0	48	2.8	20	26	18	1.7	28.2	G-2
266	0	45	0	27	7.5	16	40	18	2	33.6	G-2
267	0	37	0	33	4.7	10	24	11	3.3	33.5	G-2
268	1	70	0	66	2.5	22	44	16	3.3	42.8	G-2
269	1	47	1b	20	11	30	44	21	3.3	60	G-2
270	1	46	1b	15	6.8	10	42	10	2.7	54.6	G-2
70	28	47	36	32.1	3.4	17	33	19	1.7	48.7	
	42	14	34	22.2	2.7	7.5	9.4	6.3	1	14.9	

345	0	31	1	48	1.4	28	44	17	1	40	G-3
346	0	73	0	68	3.1	15	44	20	1	46.3	G-3
347	1	37	0b	56	0.5	24	42	22	2.5	32.5	G-3
348	0	60	0b	35	15	28	42	31	3	41.3	G-3
349	1	71	0	44	9.2	10	46	24	0.5	61.3	G-3
350	0	48	0	40	6.3	11	30	18	0.5	48	G-3
351	0	26	1	6	3.4	16	22	14	0.5	36	G-3
352	0	41	1	35	3.8	18	48	23	0.5	38.8	G-3
353	0	40	0b	60	4.1	14	50	24	0.5	41.3	G-3
354	1	46	1	42	4.2	10	36	25	2.5	58.75	G-3
355	1	72	1b	48	1.5	5	24	18	3.5	60	G-3
356	0	59	1	40	1	28	28	12	3.3	61.3	G-3
357	0	52	0	36	0.8	5	32	20	1	58.75	G-3
358	1	45	1	3	2.9	25	34	10	1	32	G-3
359	0	74	1	50	4.8	22	38	26	0.5	56	G-3
360	0	22	0	3	1.4	28	24	15	0.5	25	G-3
361	1	66	1	27	3.1	12	25	30	2	55	G-3
362	0	34	1	10	3.4	10	28	16	2	50	G-3
363	0	48	1	6	1.1	5	36	13	2	55	G-3
63	22	51.7	32	37	3.9	17	35	20	1.8	49.5	
	41	14.7	31	21	2.7	7.2	9.6	6.4	1.1	13.2	

	sex	age	lat	dur	avp	crt	sds	srs	vf	hl	
401	0	29	0b	6	2.9	22	42	18	1.3	68.8	G-4
402	1	43	1	14	4.8	12	25	30	1.3	46.7	G-4
403	1	39	0b	9	1.7	15	24	24	2	28	G-4
404	0	55	0	10	9.3	8	30	11	0.5	50	G-4
405	0	37	0	12	4.4	12	32	12	1.7	66	G-4
406	1	63	1	9	1.5	15	45	14	0.5	31.3	G-4
407	1	39	0	20	3	20	48	15	3.3	68	G-4
408	0	49	1	12	1.3	8	38	17	1	55.7	G-4
409	0	71	1	30	2.6	14	22	10	2.5	48.8	G-4
410	0	47	0b	35	5.9	19	24	22	0.5	60	G-4
411	0	58	1	45	1.1	26	33	12	0.5	43.8	G-4
412	1	73	0b	36	1.8	30	50	24	0.5	73.8	G-4
413	1	59	1	45	2	12	48	16	1	50	G-4
414	0	45	1b	70	1.4	14	50	37	1	37.5	G-4
415	0	43	0	36	1.3	15	46	20	0.5	62.5	G-4
416	1	62	1	10	3.8	10	24	24	1.7	23.8	G-4
417	0	53	1	40	0.9	22	28	25	2	61.3	G-4
418	1	64	1	110	1.4	25	32	13	2.7	68	G-4
419	0	63	0	36	3	10	34	12	0.5	22.5	G-4
420	0	55	0	36	12	12	38	30	1.7	77.5	G-4
421	0	39	1	48	5.9	25	44	16	1.7	37.5	G-4
422	0	56	0	27	16	20	25	17	2.5	63.8	G-4
423	0	53	1	33	2.9	10	28	18	1.7	40	G-4
424	1	44	1	66	12	5	24	22	3.3	66.7	G-4
425	0	42	1	20	0.8	6	45	13	1	75	G-4
426	0	41	0	15	1.7	18	22	14	2.5	28.2	G-4
427	1	73	0b	38	2.7	22	42	18	3	33.6	G-4
428	1	43	1b	66	6.8	12	25	14	0.5	55	G-4
429	0	43	1	50	2.2	28	24	16	0.5	63.8	G-4
430	0	33	1	4	0.8	15	30	10	3.3	38.8	G-4
431	1	34	1	45	2	24	32	26	1.7	30	G-4
432	0	65	0	100	1.5	28	45	12	2.3	40.5	G-4
433	1	47	0	48	1.4	10	48	10	1.7	40.3	G-4
434	1	53	1	80	1.8	11	38	23	1	40.5	G-4
435	0	63	0	38	4.4	16	22	12	2.3	28.8	G-4
436	1	47	1	20	2.6	18	24	25	2	45	G-4
437	0	29	1	38	1	14	33	22	0.5	56.3	G-4
438	1	23	1	3	1.2	10	22	20	1.7	32	G-4
439	0	48	0	42	4.2	12	48	12	0.5	47.5	G-4
440	0	45	1b	48	1.4	28	50	32	1.3	66.3	G-4
441	0	62	0	35	1.7	10	26	12	1	61	G-4
442	0	58	1	60	3.2	25	24	11	2.5	42.5	G-4
443	0	55	1	3	1.5	22	28	20	0.5	46	G-4
444	0	61	0	48	11	16	32	12	0.5	73.8	G-4

445	0	76	1	66	3	8	34	22	0.5	36.3	G-4
446	0	34	0	50	1	10	38	22	2.5	76.3	G-4
447	0	43	1b	25	3.4	24	44	21	3.5	51.3	G-4
448	0	63	0	49	2.3	25	25	30	3.3	43.5	G-4
449	1	39	0	4	1.9	18	28	23	1	32	G-4
450	1	58	1	40	1.4	10	36	15	1	70	G-4
451	0	46	1	20	6.7	14	28	18	0.5	53.4	G-4
452	1	42	0	12	2.9	22	36	24	2	62.6	G-4
453	1	45	0	30	3.8	16	48	25	1.5	38.8	G-4
454	0	64	0	35	0.9	25	50	26	2	40.3	G-4
455	0	45	0	4	1.4	30	22	12	0.5	30	G-4
456	0	49	0	10	4.5	33	23	14	2.5	28.8	G-4
457	0	45	1	33	1	25	40	24	3.5	36.3	G-4
458	0	55	1	25	5.9	22	22	17	3.3	46	G-4
459	1	37	0b	12	0.8	6	55	16	4	51.3	G-4
460	1	41	1	18	2.9	8	30	19	4	55	G-4
60	22	49.7	32	34	3.3	17	34	19	1.7	49.1	
	38	11.9	28	23	3.1	7.2	9.8	6.3	1.1	15.1	
